# Computed Tomography-Verified Deep Learning Model for Rib Fracture Prediction and Complication Association from Chest Radiographs

**DOI:** 10.7150/ijms.131105

**Published:** 2026-07-22

**Authors:** Ting-Hsuan Chao, Chih-Chien Wang, Chin Lin, Chin-Sheng Lin, Teng-Wei Chen, Shih-Hua Lin, Dung-Jang Tsai

**Affiliations:** 1 Department of Orthopedic Surgery, Tri-Service General Hospital, National Defense Medical University, Taipei, Taiwan, R.O.C.; 2 Medical Technology Education Center, School of Medicine, College of Medicine, National Defense Medical University, Taipei, Taiwan, R.O.C.; 3 Military Digital Medical Center, Tri-Service General Hospital, National Defense Medical University, Taipei, Taiwan, R.O.C.; 4 Division of Cardiology, Department of Internal Medicine, Tri-Service General Hospital, National Defense Medical University, Taipei, Taiwan, R.O.C.; 5 Division of General Surgery, Department of Surgery, Tri-Service General Hospital, National Defense Medical University, Taipei City, 114, Taiwan, R.O.C.; 6 Division of Nephrology, Department of Internal Medicine, Tri-Service General Hospital, National Defense Medical University, Taipei, Taiwan, R.O.C.

**Keywords:** artificial intelligence, chest X-ray, rib fracture, deep learning model, pneumothorax, pulmonary contusion

## Abstract

**Background:**

Chest radiography (CXR) is the first-line imaging for suspected rib fractures but has limited sensitivity, potentially increasing the risk of thoracic complications. This study aimed to develop and validate a deep learning model (DLM) based on CT-verified labels to predict acute rib fractures on CXRs and evaluate its association with thoracic complications.

**Methods:**

This retrospective study included 14,758 patients who underwent chest CT and CXR within seven days. We used 10,818 CXRs for development, 4,377 for tuning, and 3,020 for validation. The DLM employed a Vision Transformer (ViT-B/32) architecture. Performance was assessed using AUC, sensitivity, and predictive values. Risk stratification was performed via Youden index and F-score optimization.

**Results:**

The DLM achieved an AUC of 0.874, sensitivity of 84.9%, and negative predictive value (NPV) of 99.4% in validation. Performance remained consistent across care settings (AUCs 0.819-0.853). Subgroup analysis showed the best performance in patients < 60 years (AUC 0.934), while comorbidities were associated with lower AUCs. High-risk patients exhibited significantly higher one-month and one-year incidences of pneumothorax (17.4% and 12.5%) and pulmonary contusion (9.8% and 8.0%) compared to low-risk patients (all p < 0.001). No significant difference was observed for pneumonia association.

**Conclusions:**

This CT-verified DLM demonstrated high diagnostic performance for predicting acute rib fractures and effectively stratified patients by associated complication risk. This model may assist clinicians with early prediction and triage, potentially enhancing clinical efficiency and patient outcomes in trauma workflows.

## Introduction

Rib fractures are a major global health issue in trauma care and have substantial clinical and economic implications. According to the Global Burden of Disease Study in 2019, the worldwide incidence of rib fractures was approximately 4.1 million cases, reflecting a 43.7% increase since 1990 and an age-standardized incidence rate (ASIR) of 52.2 per 100,000 person-years 1. In Taiwan, rib fractures occur in 40-80% of blunt thoracic trauma cases, as demonstrated by a retrospective cohort study of 1,621 patients at a level I trauma center. The overall mortality rate is 6.9%, and 11.7% of patients with severe injury (Injury Severity Score [ISS] ≥ 16) require intensive care. Motorcycle accidents are associated with 2-3-fold greater risk of multiple rib fractures in unhelmeted riders, compounded by frequent concurrent injuries, such as hemothorax (31.8%), pneumothorax (15.6%), and hemopneumothorax (4.6%). Notably, elderly patients (≥ 65 years) experience prolonged hospitalization and higher complication rates, emphasizing age as a critical risk factor^2^.

From a clinical perspective, rib fractures are commonly associated with significant thoracic complications, particularly pneumothorax, pulmonary contusion, and pneumonia. Studies report that pneumothorax occurs in approximately 10-15% of patients with rib fractures and is often linked to multiple or displaced fractures^3^. Pulmonary contusions are present in 19-30% of cases, with bilateral contusions significantly increasing the risk of respiratory failure and mortality^4^, ^5^. Pneumonia develops in 8-15% of patients, particularly among elderly individuals, heavy drinkers, and those with multiple rib fractures^6^, ^7^. The number of fractured ribs, patient age, comorbidities such as chronic obstructive pulmonary disease, and the severity of initial trauma are important predictors of these complications. Early detection and management of rib fractures are critical to prevent progression to severe outcomes. Therefore, the use of advanced imaging techniques or adjunctive tools may substantially reduce the morbidity and mortality associated with thoracic trauma.

Chest radiography (CXR) remains the initial imaging modality for evaluating suspected rib fractures because of its accessibility, low cost, and relatively low radiation exposure. However, the sensitivity of CXR for detecting rib fractures is limited, particularly for nondisplaced fractures and those involving the costal cartilages^8^. Up to 50% of rib fractures may be missed on standard CXR^9^. Studies have demonstrated that even experienced radiologists may miss a substantial proportion of rib fractures on initial imaging^10^. A recent study developed a deep learning model for detecting rib fractures on chest radiographs, achieving an area under the ROC curve (AUROC) of 0.89 and a Jackknife Alternative Free-response Receiver Operating Characteristic (JAFROC) figure of merit of 0.76, demonstrating performance comparable to that of radiologists. These findings highlight the potential of AI-assisted tools to increase diagnostic accuracy and efficiency in clinical practice^11^.

Computed tomography (CT) has emerged as the reference standard for detecting rib fractures^12^, offering higher sensitivity and the added benefit of identifying associated injuries. Nonetheless, its application is restricted because of increased radiation exposure, which is of particular concern in pediatric and pregnant populations, along with increased costs and limited accessibility in certain health care systems. These limitations underscore the need for improved diagnostic methods that maintain the advantages of CXR while approaching the diagnostic accuracy of CT.

To address the diagnostic challenges associated with rib fractures on CXR, we developed an artificial intelligence (AI) model trained on rib fractures confirmed by CT findings. An AI model that predicts CT-confirmed rib fractures from CXR could serve as a triage tool to prioritize CT scanning, reduce missed injuries in resource-limited or high-throughput emergency departments, and support early risk stratification. This model aims to assist clinical practitioners—including emergency physicians, nonradiologist physicians, and junior physicians—in accurately detecting rib fractures on CXR. By providing a diagnostic performance approaching that of radiology specialists, the AI tool seeks to enhance early fracture detection, particularly in high-pressure clinical settings and among nonradiologist physicians. Early identification of rib fractures is critical for reducing associated complications, such as pneumothorax and pneumonia, thereby potentially lowering mortality rates and improving patient outcomes.

## Methods

### Data source and study population

This multi-site, retrospective cohort study was conducted at Tri-Service General Hospital (Taipei, Taiwan) and its affiliated Tingjhou branch. We received ethical approval from the Institutional Review Board (IRB No. C202305019). CXR data were collected from the general population, including outpatient, emergency department, and inpatient settings, between January 1, 2011, and February 28, 2022. Rib fracture was diagnosed using chest CT, and the diagnosis was confirmed. All patients included in the study were required to have undergone at least one CXR and one chest CT examination within 7 days of each other on the basis of clinical indications. Individuals younger than 18 years were excluded.

**Figure 1:**
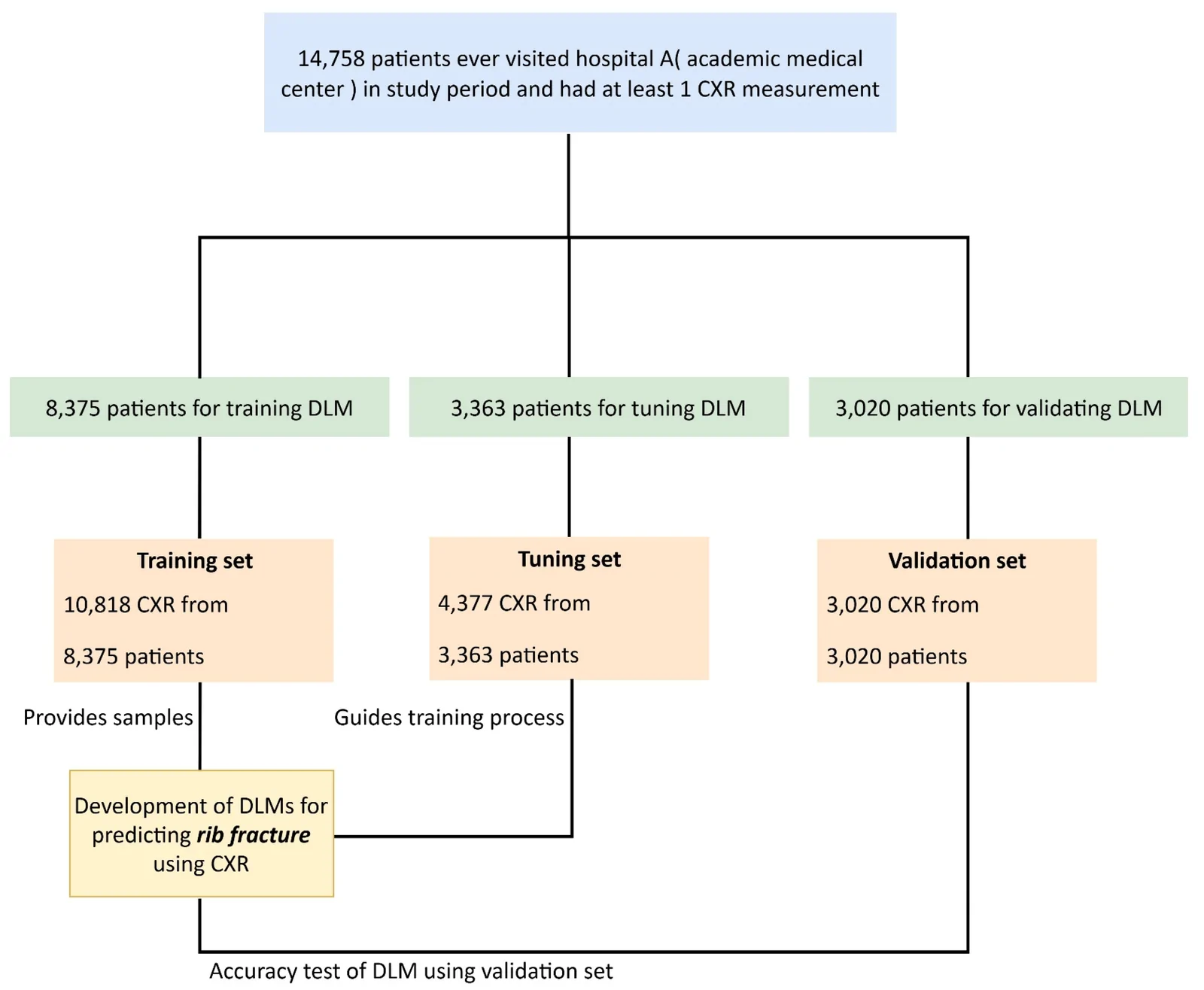
** Generation of datasets for training, tuning and validation.** Schematic of the dataset creation and analysis strategy, which was devised to ensure a robust and reliable dataset for the training and tuning of the network. Once a patient's data were placed in one of the datasets, that individual's data were used only in that set, avoiding “cross-contamination” among the training, tuning, and validation datasets. The details of the flowchart and how each dataset was used are described in the Methods section.

A total of 14,758 patients with at least one CXR were included in the study (Figure 1). These CXR data were randomly divided into three datasets, which were stratified on the basis of key demographic and clinical variables (age, sex, and comorbidities) to ensure representative sampling and minimize selection bias. Approximately 10,818 CXRs from 8,375 patients were used for model development, 4,377 CXRs from 3,363 patients were used for model tuning, and 3,020 CXRs from 3,020 patients from the Tingjhou branch, a regional hospital, were used for validation. The aim of this study was to develop a model for detecting rib fractures from CXRs.

### Variables

#### Comorbidity classification

Preexisting medical conditions were systematically categorized using standardized International Classification of Diseases (ICD) codes across both the ICD-9 and the ICD-10 systems (Table S1). The conditions included pneumothorax, rib fracture, pneumonia, lung contusion, diabetes mellitus (DM), hypertension (HTN), hyperlipidemia (HLP), chronic kidney disease (CKD), heart failure (HF), coronary artery disease (CAD), and chronic obstructive pulmonary disease (COPD).

#### Radiographic feature annotation

Each chest radiograph underwent comprehensive analysis through a standardized annotation protocol incorporating 45 distinct radiological parameters. This systematic approach utilized advanced natural language processing algorithms combined with expert radiologist validation to ensure data integrity. The annotation framework categorized findings across multiple anatomical systems: respiratory pathology (pneumonic consolidation, emphysematous destruction, pneumothorax, atelectatic changes, pulmonary vascular congestion), pleural space abnormalities (diaphragmatic contour irregularities, costophrenic angle obliteration, pleural fluid accumulation), cardiac and vascular manifestations (atherosclerotic calcification, cardiac chamber enlargement, hilar prominence, vascular dilation), skeletal pathology (arthritic changes, traumatic injuries, spinal degeneration, bone density alterations), mediastinal alterations (compartment widening), neoplastic processes (malignant lesions, inflammatory responses), and therapeutic interventions (drainage systems, surgical modifications, implanted devices, airway management equipment, nutritional support apparatus). This comprehensive labeling methodology provides the analytical foundation for the AI-driven radiographic interpretation platform.

### Study outcomes

Ground-truth labels for rib fracture status were established through a systematic review of structured chest computed tomography reports using predefined rule-based query prompts, with the original prompt wording incorporated directly into the outcome definition to ensure transparency and reproducibility. Three binary queries were applied to each report: (Q1) whether any rib fracture was mentioned, (Q2) whether an old rib fracture was described, including callus formation, and (Q3) whether both an old rib fracture (including callus formation) and a nonold rib fracture were described separately in different sections of the report, with callus formation explicitly excluded from the nonold fracture description. Cases of acute rib fracture were defined as reports with Q1 = yes, Q2 = no, and Q3 = no, indicating the presence of a rib fracture without radiological features of chronicity, as well as reports with Q1 = yes, Q2 = yes, and Q3 = yes, indicating the coexistence of an old rib fracture and a newly described rib fracture within the same report, which confirmed the presence of an acute fracture despite concomitant chronic findings. Reports with Q1 = no were classified as nonfracture cases and served as the control group. This structured rule-based extraction approach enabled consistent and reproducible identification of acute rib fracture outcomes, including clinically relevant scenarios in which acute and chronic rib fractures coexisted. To ensure the reliability of this automated labeling process, a clinician (lead author) conducted a pilot manual validation by randomly reviewing 100 CT reports. This audit demonstrated 100% concordance between the manual clinical interpretation and the NLP-generated labels, confirming the accuracy of the rule-based ground-truth extraction before its application to the entire study population.

### Implementation of the deep learning and machine learning models

Radiographic data were acquired and maintained in the Digital Imaging and Communications in Medicine (DICOM) standard format, with native resolution parameters exceeding 2000 × 2000 pixels. The foundational neural network architecture employed the CheXzero methodology^13^, incorporating dual-encoder systems comprising visual and textual processing components trained on paired radiograph-report datasets. For this investigation, we exclusively utilized the visual encoding module, implementing a Vision Transformer architecture (ViT-B/32 configuration) for radiographic feature extraction.

Image preprocessing involved standardized resizing to 256 × 256 pixels to accommodate network input requirements, followed by pixel intensity normalization to maintain uniform data distribution characteristics^14^. The encoding process generated 512-dimensional feature representations for each radiographic examination. The pretrained weights “best_64_0.0001_original_35000_0.864” were used without further adjustment. To estimate the probability of rib fracture, logistic regression models were fitted with the AI-CXR feature vectors as inputs. Risk stratification protocols categorized patients into tertiles (low-, median-, and high-risk) on the basis of model-generated probability scores. Optimal classification boundaries were determined through Youden index optimization and F-score maximization techniques applied to validation cohort performance metrics for rib fracture. The computational implementation utilized the Python programming environment (version 3.10.10) with the PyTorch deep learning framework (version 2.0.1).

### Statistical analysis

Baseline characteristics were summarized using means with standard deviations for continuous variables and frequencies with percentages for categorical variables. Continuous variables were compared using independent Student's t tests or analysis of variance (ANOVA), as appropriate. Categorical variables were analyzed using chi-square tests or Fisher's exact tests according to expected cell counts. The primary endpoint was the prediction of CT-confirmed acute rib fracture. A logistic regression model was fitted using the 512-dimensional CXR feature vectors extracted by the Vision Transformer encoder as predictors. Model discrimination was evaluated using the area under the receiver operating characteristic curve (AUC). Diagnostic performance was quantified by accuracy, sensitivity, specificity, positive predictive value, and negative predictive value in the validation cohort. Risk stratification into low-, median-, and high-risk categories was derived from the predicted probabilities using the Youden index and the F-score determined in the tuning cohort. Secondary analyses focused on the occurrence of pneumothorax, pulmonary contusion, and pneumonia. Kaplan-Meier survival curves were used to describe the cumulative incidence of these complications across the CXR-predicted risk strata. The log-rank test was used to assess statistical significance. The deep learning model did not predict survival or time to event. Kaplan-Meier methods were used only to illustrate the association between model-derived risk groups and subsequent clinical events. For the one-year analyses, cases with outcomes recorded within the first 30 days were excluded to reduce potential misclassification related to ICD-based coding practices. Statistical analysis was conducted using R software version 3.4.4. Two-sided p values less than 0.05 were considered to indicate statistical significance.

## Results

### Baseline characteristics

The baseline characteristics of the patients in the training, tuning, and validation cohorts are presented in Table 1. The mean age was comparable across the three cohorts, with 64.2 ± 18.6 years in the training cohort, 64.5 ± 18.6 years in the tuning cohort, and 59.9 ± 18.5 years in the validation cohort. The sex distribution was similar, with males constituting approximately 57-58% across all groups. More than half of the patients in the training and tuning cohorts were from the emergency room (ER, 52.7% and 53.5%, respectively); moreover, in the validation cohort, more patients were from the outpatient department (OPD, 39.1%). In the training cohort, 22.2% of patients had DM, which was comparable to the 22.6% in the tuning cohort but decreased to 15.0% in the validation cohort (p < 0.001). HTN was present in 7.7%, 8.1%, and 4.1% of patients in the training, tuning, and validation sets, respectively (p < 0.001). The prevalence of CKD was 23.8% in the training cohort, 23.2% in the tuning set, and 10.7% in the validation set, with a significant difference across datasets (p < 0.001). Hyperlipidemia (HLP) was observed in 27.5%, 28.0%, and 21.6% of the patients, respectively (p < 0.001). Heart failure (HF) occurred in 11.0% of the training set, 12.4% of the tuning set, and 5.7% of the validation set (p < 0.001). Similarly, CAD was identified in 21.3%, 22.3%, and 15.1% of the respective datasets (p < 0.001). The prevalence of COPD was 17.8% in the training cohort, 19.0% in the tuning set, and 16.7% in the validation set (p = 0.054), with no statistically significant difference among the groups. Acute rib fracture-related risk stratification was similar across the three sets, with the majority classified as low risk (84.9%, 85.9%, and 90.4% in the training, tuning, and validation cohorts, respectively). Only a small fraction were identified as high risk (4.2%, 3.9%, and 2.6%). Among all patients, the proportions of those with an actual diagnosis of acute rib fracture were 4.6%, 4.5%, and 2.8% in the training, tuning, and validation sets, respectively (p < 0.001). When the data were stratified by the CXR-predicted rib fracture risk groups, a clear gradient relationship was observed between the predicted fracture probability and actual complication incidence. In the low-risk group, only 1.2% of patients were confirmed to have an acute rib fracture. In contrast, the incidence increased markedly to 10.3% in the median-risk group and further rose to 38.5% in the high-risk group (p < 0.001).

### Performance of DLM to identify acute rib fracture

**Figure 2:**
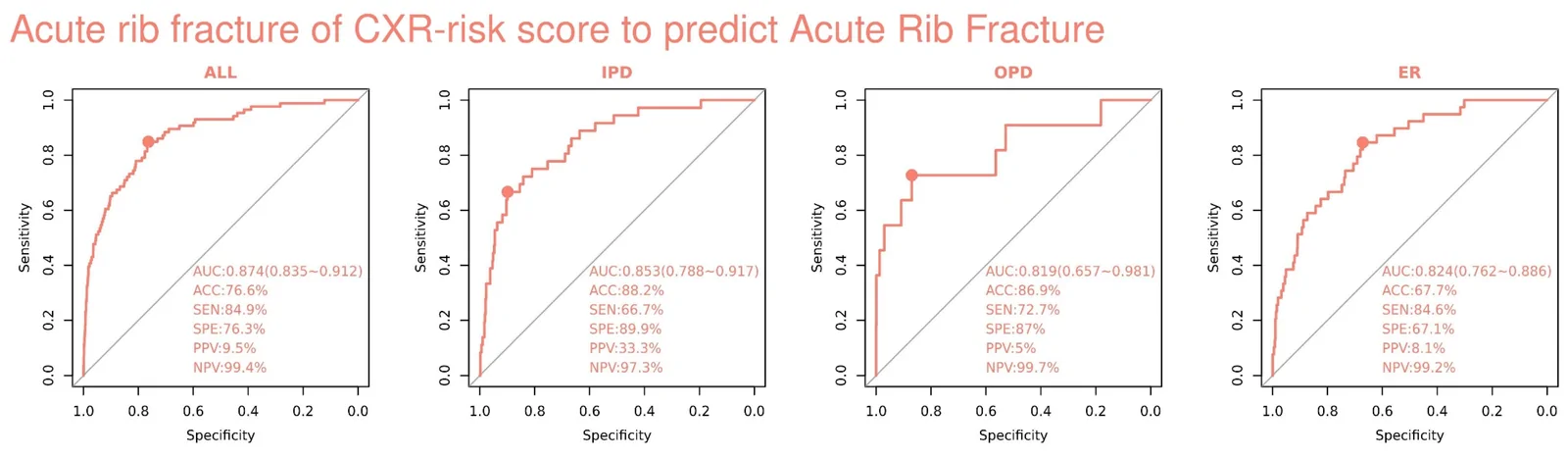
** Receiver operating characteristic (ROC) curve of deep learning model (DLM) predictions based on chest radiography (CXR) for detecting acute rib fracture.** Performance of the CXR risk score in risk stratification. The receiver operating characteristic curve and area under the curve (AUC) with 95% confidence intervals, accuracy (ACC), sensitivity (SEN), specificity (SPE), positive predictive value (PPV), and negative predictive value (NPV) of acute rib fracture according to the CXR risk score in the validation dataset.

**Figure 3:**
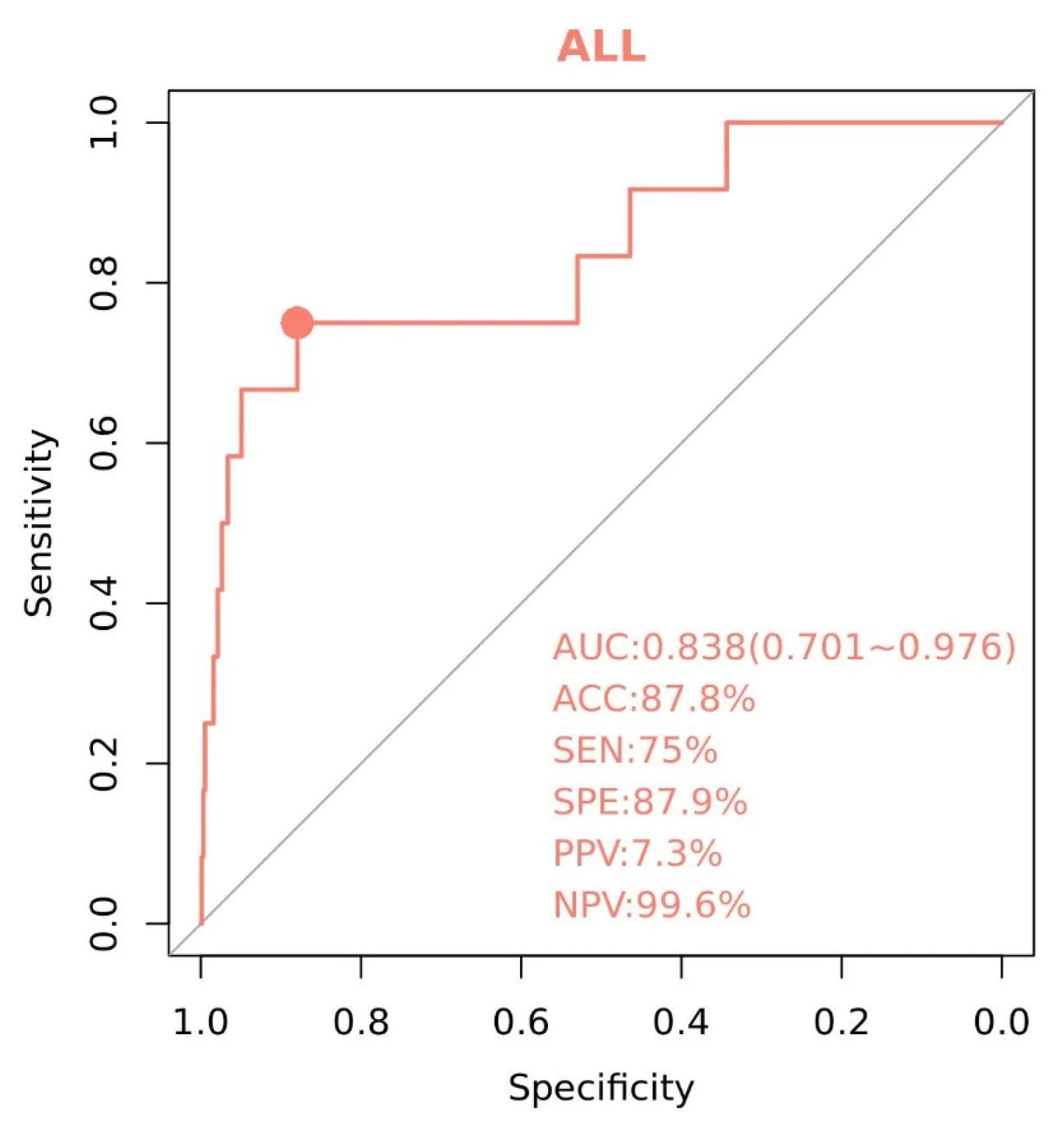
** Ablation test of deep learning model (DLM) predictions based on chest radiography (CXR) for detecting acute rib fracture, after excluding images with life-support tubes and nasogastric tubes.** Performance of the CXR risk score in ablation and stratified analysis after excluding images with life-support tubes and nasogastric tubes. The receiver operating characteristic curve and area under the curve (AUC) with 95% confidence intervals, accuracy (ACC), sensitivity (SEN), specificity (SPE), positive predictive value (PPV), and negative predictive value (NPV) of acute rib fracture.

To predict acute rib fracture, the deep learning model (DLM) achieved an overall AUC of 0.874(CI: 0.84-0.91), with an accuracy of 76.6%, sensitivity of 84.9%, specificity of 76.3%, positive predictive value (PPV) of 9.5%, and negative predictive value (NPV) of 99.4% in the validation dataset (Figure 2). Stratified by hospital department, the model performed in the inpatient department (IPD) had an AUC of 0.853(CI: 0.79-0.92), an accuracy of 88.2%, and a specificity of 89.9%, although the sensitivity was relatively low (66.7%). In the outpatient department (OPD) set, the model achieved an AUC of 0.819(CI: 0.66-0.98), with an accuracy of 86.9% and a specificity of 87.0%. For emergency room (ER) cases, the AUC was 0.824(CI: 0.76-0.89), with a sensitivity of 84.6% and an NPV of 99.2%. To assess potential confounding by clinical severity markers, an ablation analysis was conducted on a subset of radiographs without medical tubes or devices. The model achieved an AUC of 0.838(CI: 0.701-0.976), indicating that the predictive performance was sustained by intrinsic radiographic features of the ribs rather than shortcut indicators of intubation or ICU-level care (Figure 3).

**Figure 4:**
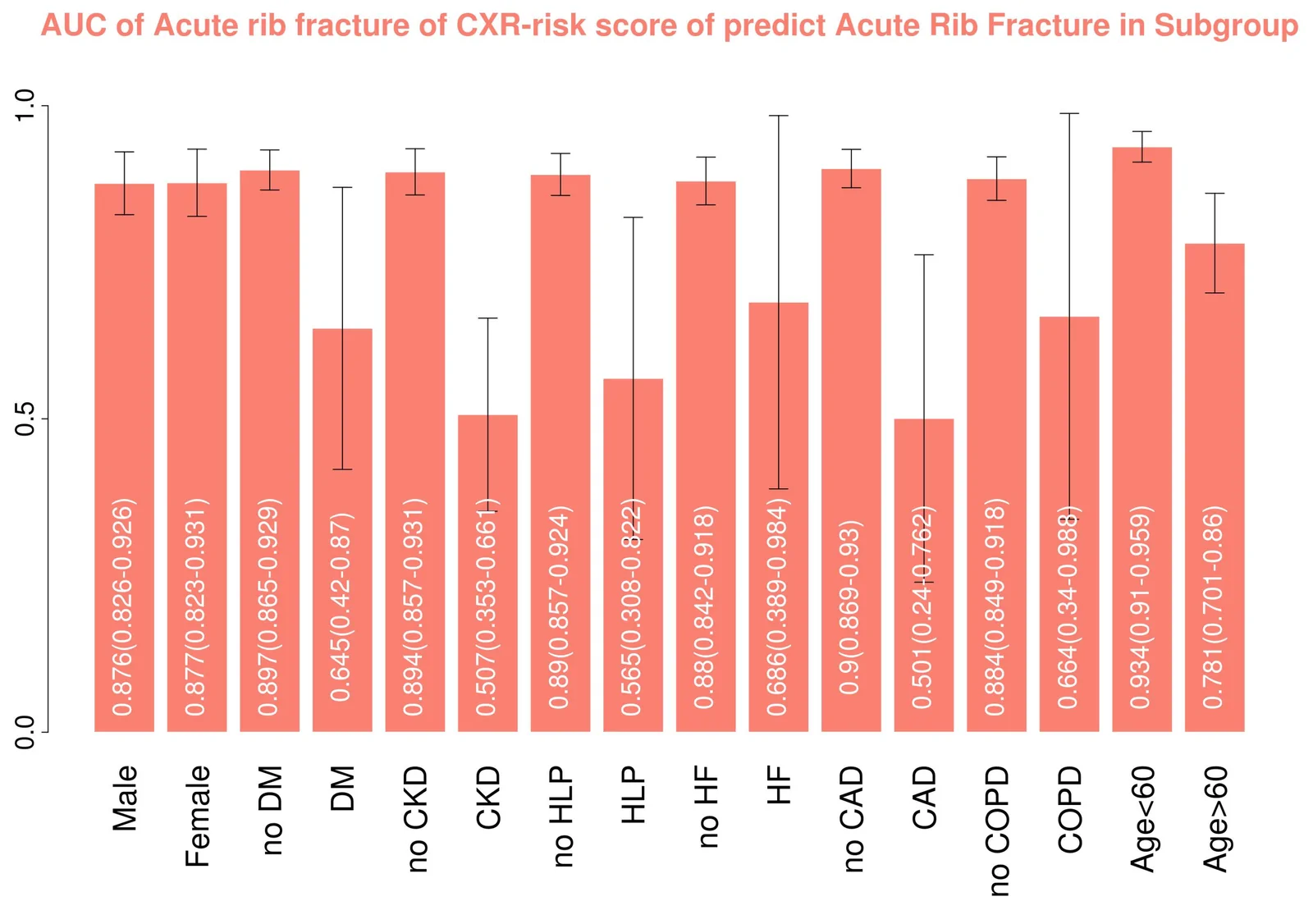
** Area under the curve (AUC) of deep learning model (DLM) predictions based on chest radiography (CXR) for detecting acute rib fracture and rib fracture by subgroup.** Stratified analysis of the AUC with 95% confidence intervals in different subgroups for acute rib fracture and rib fracture. Abbreviations: Gender, (male and female); DM, diabetes mellitus; CKD, chronic kidney disease; HLP, hyperlipidemia; HF, heart failure; CAD, coronary artery disease; COPD, chronic obstructive pulmonary disease; age, >60 years and <60 years.

Further subgroup analyses are shown in Figure 4. The AUC values for rib fracture prediction were consistent across genders, with both males (AUC 0.876, 95% CI: 0.826-0.926) and females (AUC 0.877, 95% CI: 0.823-0.931) achieving AUCs of approximately 0.88-0.90. Patients without DM had a greater AUC (0.897, 95% CI: 0.865-0.929) than those with DM did (0.645, 95% CI: 0.42-0.87). Similarly, those without CKD had better performance (AUC 0.894, 95% CI: 0.857-0.931) than those with CKD did (AUC 0.507, 95% CI: 0.353-0.661). The presence of HF and CAD was also associated with decreased AUCs (0.686, 95% CI: 0.389-0.984 and 0.501, 95% CI: 0.24-0.762, respectively). In contrast, the fracture prediction performance of the HLP (AUC 0.565, 95% CI: 0.308-0.822) and COPD (AUC 0.664, 95% CI: 0.34-0.988) subgroups did not significantly decrease. When patients were stratified by age, those younger than 60 years had the highest predictive performance, with an AUC of 0.934 (95% CI: 0.91-0.959), whereas the AUC was 0.781 (95% CI: 0.701-0.86) for those older than 60 years. These findings suggest that the model has reliable predictive ability across most subgroups, although comorbidities, such as DM, CKD, HF, and CAD, negatively impact performance.

**Figure 5:**
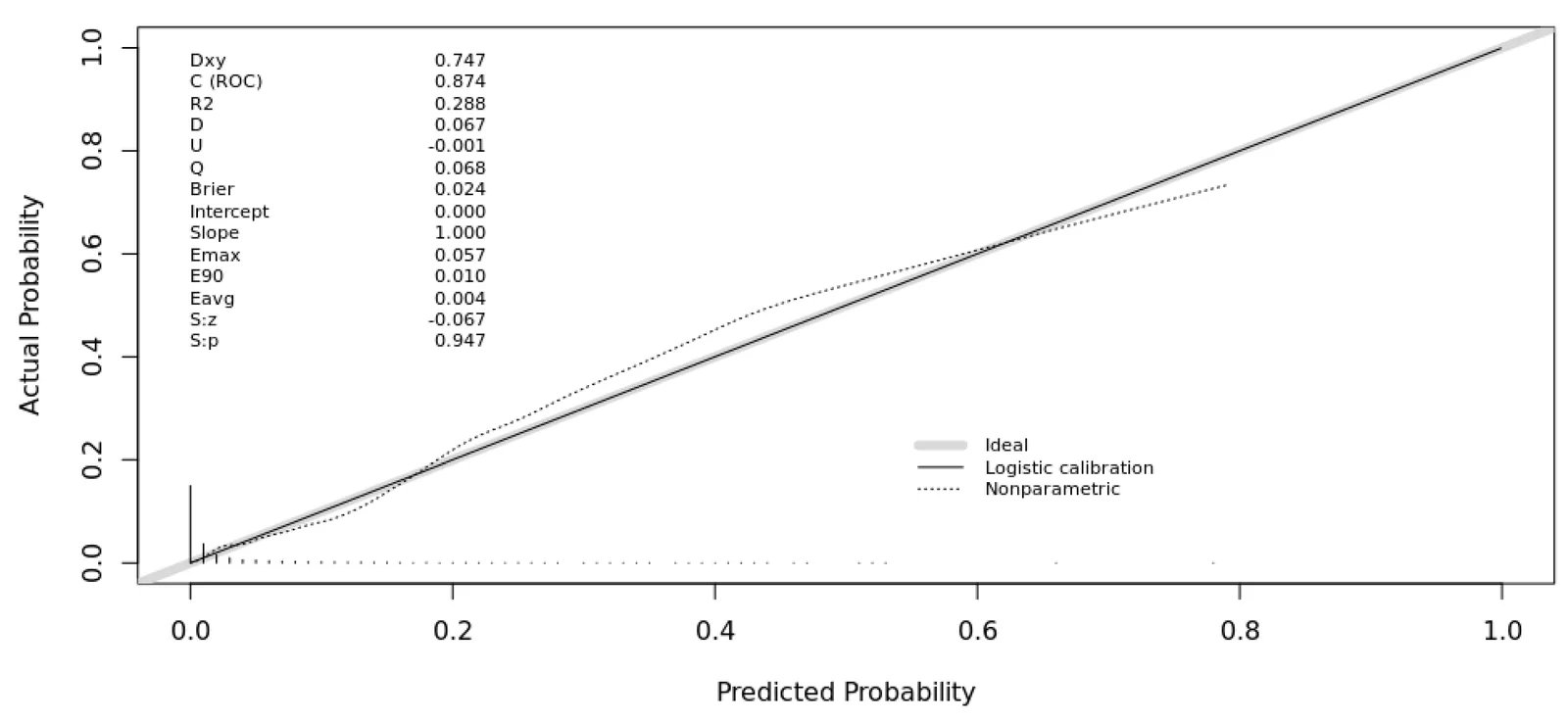
** Calibration curve for rib fracture prediction.** The x-axis represents the predicted probability generated by the model, and the y-axis represents the observed event probability. The light grey diagonal line indicates perfect calibration (ideal agreement between predicted and observed probabilities). The solid black line shows logistic calibration, while the dotted line represents the nonparametric calibration estimate. The histogram at the bottom illustrates the distribution of predicted probabilities in the dataset. The model demonstrated a C-index of 0.874, a calibration slope of 1.000, an intercept of 0.000, and a mean absolute calibration error (Eavg) of 0.004. The Brier score of 0.024 further indicates excellent predictive accuracy and strong agreement between predicted probabilities and observed outcomes.

The calibration of the deep learning model was evaluated to assess the agreement between the predicted probabilities of rib fractures and the observed outcomes (Figure 5). The calibration curve demonstrated consistent reliability, with a calibration slope of 1.000 and an intercept of 0.000, representing alignment with the ideal reference line. The mean absolute error (Eavg) was 0.004, reflecting the model's precision in probability estimation across the sampled distribution. While the concordance index (C-index) was 0.874, the Brier score of 0.024 and the minimal calibration error suggest that the model's output is appropriately calibrated for risk assessment within this cohort.

**Figure 6:**
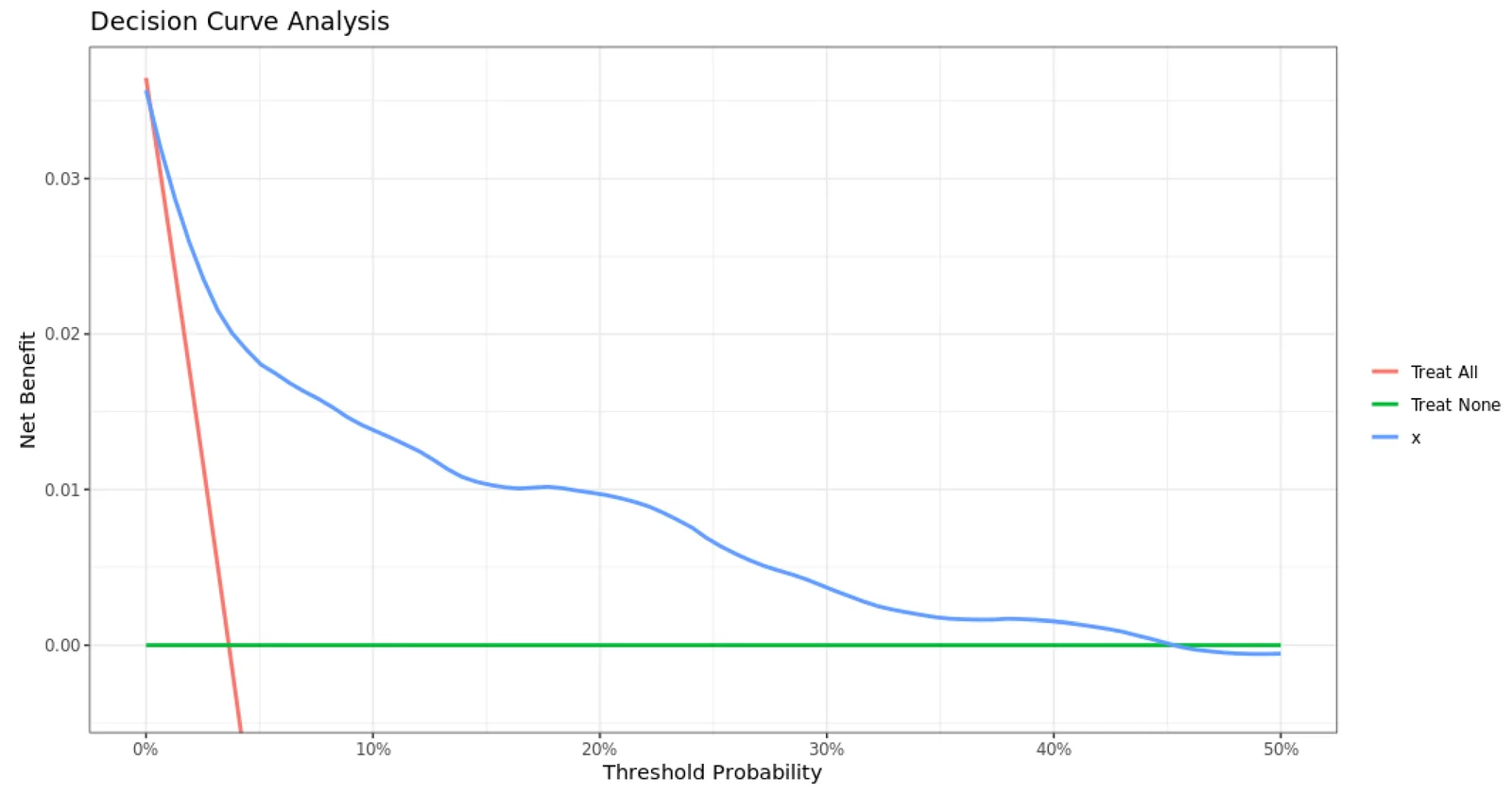
** Decision curve analysis (DCA) of the deep learning model.** The x-axis represents the threshold probability and the y-axis represents the net benefit. The blue curve shows the model's net benefit compared with the treat-all (red) and treat-none (green) strategies. The model demonstrated a positive net benefit across a threshold probability range of approximately 2% to 45%, suggesting potential clinical utility for risk stratification in rib fracture management.

Regarding clinical utility, Decision Curve Analysis (DCA) was performed to estimate the net benefit of the model across various threshold probabilities (Figure 6). The analysis indicated that the deep learning model provided an added net benefit compared to both the treat-all and treat-none strategies within a clinical threshold range of approximately 2% to 45%. This range of clinical applicability suggests that the model may serve as a supportive tool for risk stratification, potentially assisting clinicians in identifying patients who could benefit from further intervention or closer monitoring for rib fracture-associated complications.

### Association of common complications

**Figure 7:**
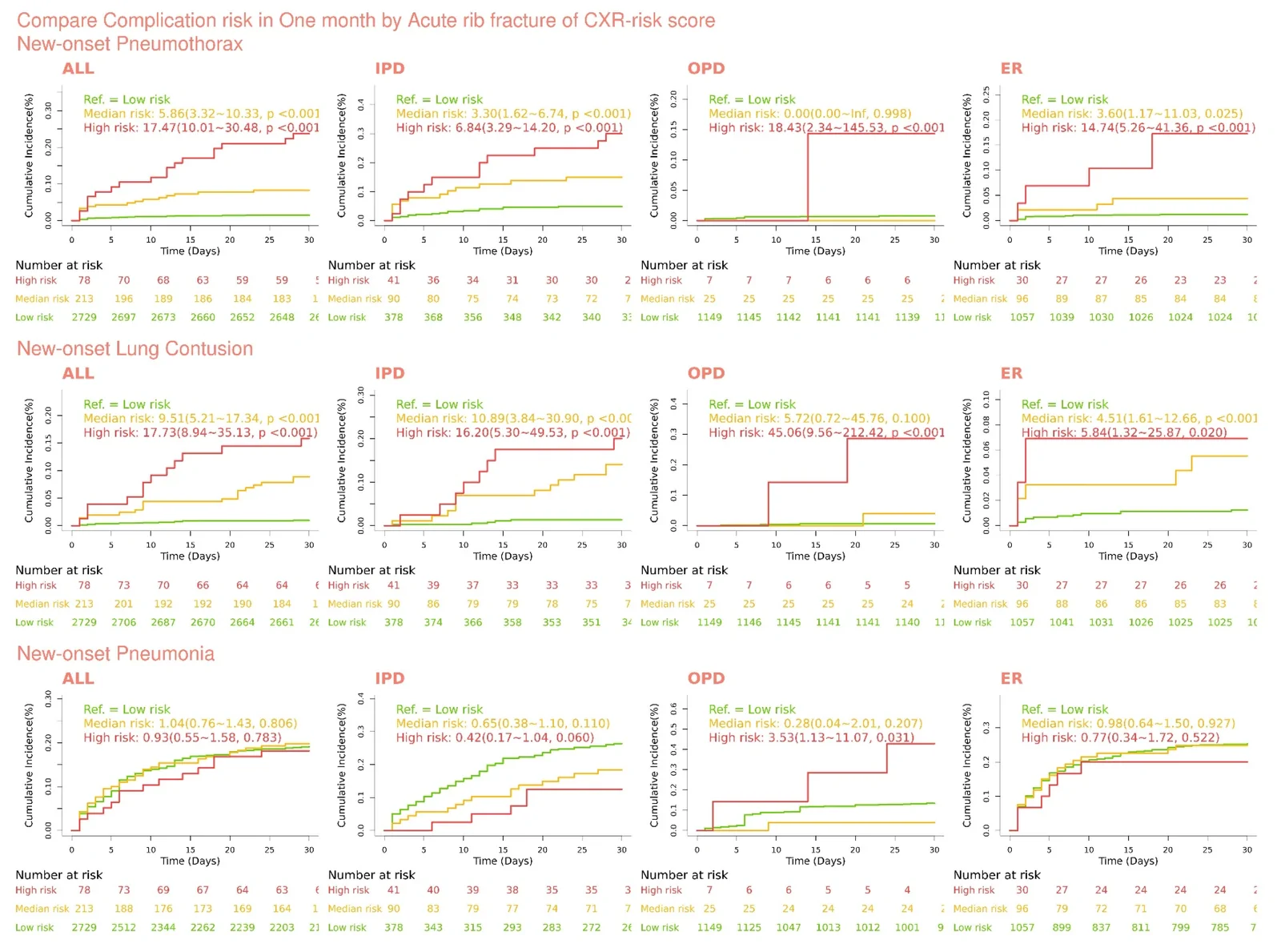
** One-month incidence of complication disease-related events stratified by chest radiography (CXR) predicted low-, median-, and high-risk acute rib fracture in the validation dataset.** Kaplan-Meier curves for one-month incidence of developing rib fracture, pneumothorax, lung contusion and pneumonia show that CXR can predict low-, median- and high-risk acute rib fracture in the validation dataset. Comparisons of the survival curves of low-, median-, and high-risk patients are shown as p values on the graph.

The one-month cumulative incidence of new-onset pneumothorax, pulmonary contusion, and pneumonia among patients stratified into low-, median-, and high-risk groups according to the CXR-predicted probability of acute rib fracture in the validation dataset is shown in Figure 7. With respect to new-onset pneumothorax, the incidence increased proportionally with the CXR-predicted risk level. Patients in the low-risk group had a cumulative incidence of 1.9% compared with 7.1% in the median-risk group and 17.4% in the high-risk group (*log-rank p* < 0.001). Similar gradients were observed across all care settings. In the inpatient cohort, the incidence increased from 0.8% in the low-risk group to 6.4% in the median-risk group and 6.9% in the high-risk group (*p* < 0.001). In the emergency department cohort, the corresponding incidences were 0.6%, 3.3%, and 13.3%, respectively (*p* < 0.001). For new-onset pulmonary contusion, a consistent stepwise increase was observed across risk strata. The low-risk group had an incidence of 0.3% compared with 3.1% in the median-risk group and 9.8% in the high-risk group (*log-rank p* < 0.001). The pattern was consistent across the inpatient and emergency cohorts (e.g., inpatients: 0.8%, 2.6%, and 12.1% for the low-, median-, and high-risk groups, respectively; *p* < 0.001). For new-onset pneumonia, however, the one-month cumulative incidence did not differ significantly among the three risk groups (low 1.8%, median 2.0%, high 1.9%, *log-rank p* = 0.118).

**Figure 8:**
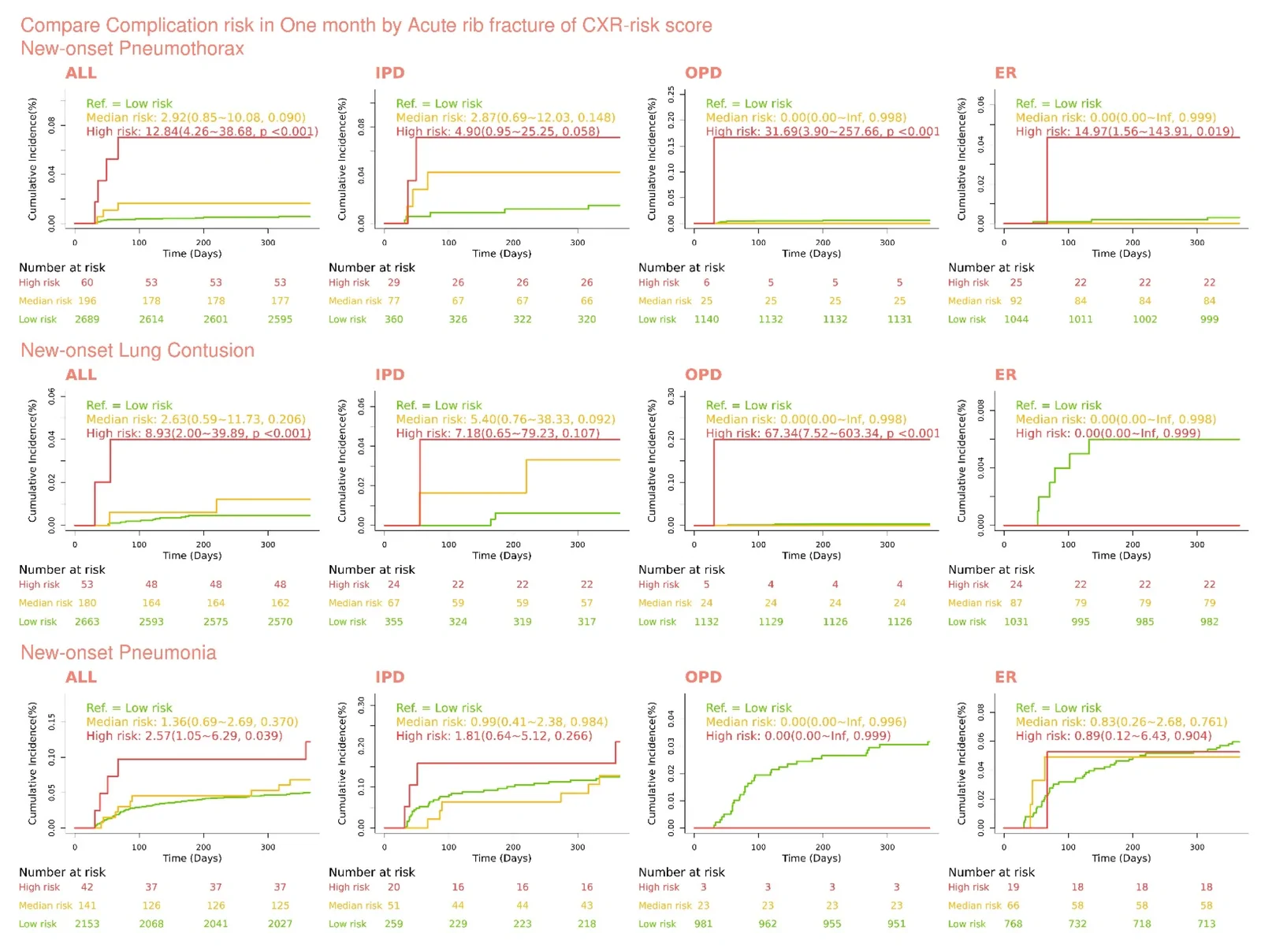
** One-year incidence of complication disease-related events stratified by chest radiography (CXR) predicted low-, median-, and high-risk acute rib fracture in the validation dataset.** Kaplan-Meier curves for one-year incidence of developing rib fracture, pneumothorax, lung contusion, and pneumonia show that CXR can predict low-, median-, and high-risk acute rib fracture in the validation dataset. Comparisons of the survival curves of low-, median-, and high-risk patients are shown as p values on the graph.

The one-year incidence of complication-related events after excluding patients who developed complications within the first 30 days is shown in Figure 8. Thirty-day events were excluded because ICD-10 coding is typically performed during hospitalization or before discharge and therefore may not reflect the exact timing of complication onset. By removing cases coded within 30 days, we aimed to exclude events occurring during the index hospitalization and instead focused on predicting future complications. With respect to new-onset pneumothorax, patients with higher CXR-predicted rib fracture risk exhibited a markedly increased one-year cumulative incidence. The low-risk group had an incidence of 1.9%, which increased to 6.3% in the median-risk group and 12.5% in the high-risk group (*log-rank p* < 0.001). Similar risk gradients were observed across all subgroups, including the inpatient (low 0.8%, median 4.9%, and high 9.0%) and emergency (low 0.6%, median 3.3%, and high 15.4%) cohorts, demonstrating the consistent descriptive association between the CXR risk score and long-term outcomes. With respect to new-onset pulmonary contusion, a stepwise relationship persisted across risk strata, although the overall incidence was lower than that observed in the one-month analysis. The one-year incidence was 0.3% in the low-risk group, compared with 2.4% in the median-risk group and 8.0% in the high-risk group (*log-rank p* < 0.001). This pattern was consistent across all care settings, reinforcing the sustained predictive value of the AI-derived CXR risk score for contusion events beyond the immediate postinjury period. In contrast, for new-onset pneumonia, no significant difference in one-year incidence was observed among the three groups (low 1.8%, median 2.5%, high 2.5%, *log-rank p* = 0.39), indicating that the predictive performance of the model for this complication was limited.

### Correlation of components

**Figure 9:**
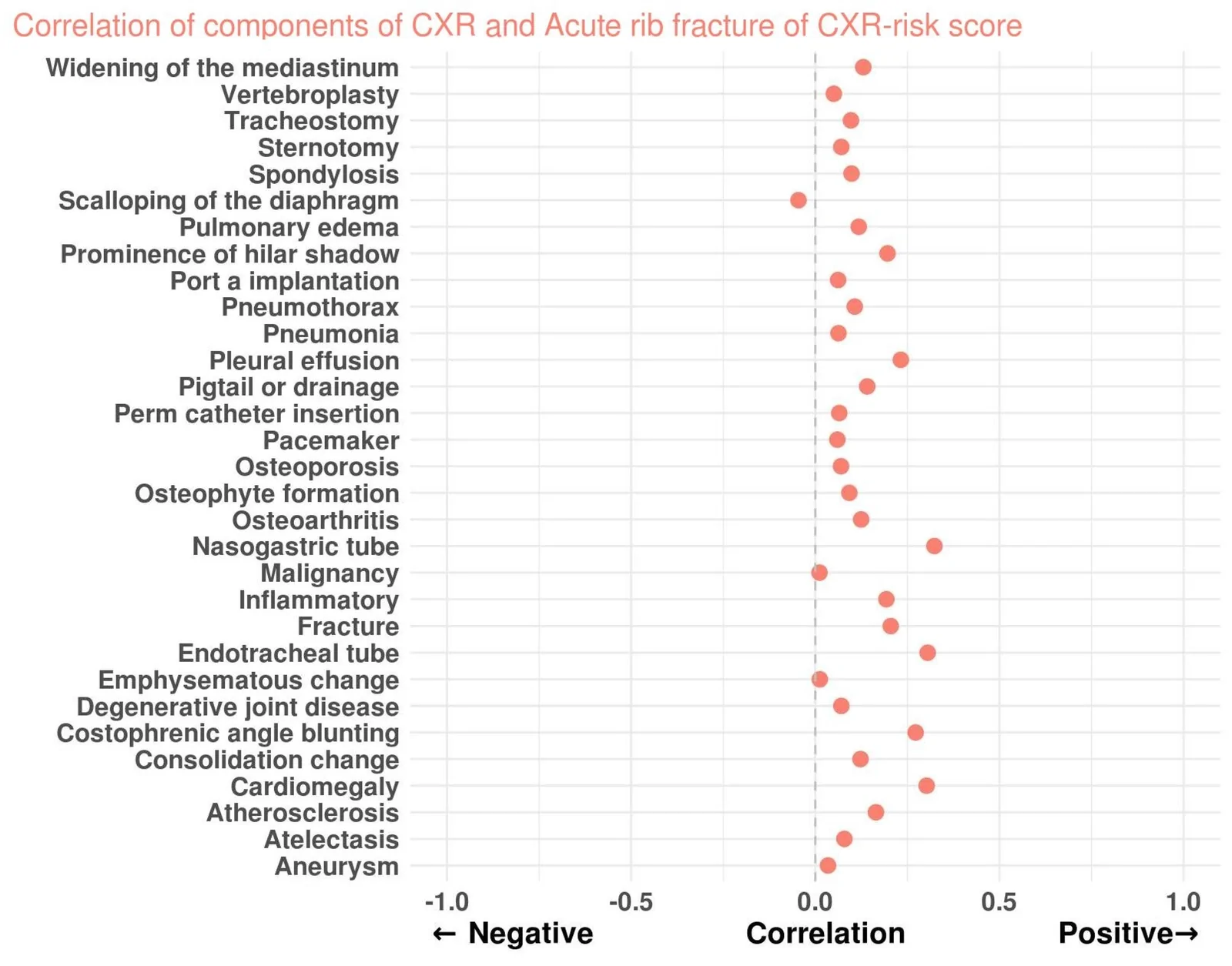
** Correlation of components of chest radiographic (CXR) and predicted acute rib fracture.** Spearman correlations were used to determine the correlation components of CXR and predicted acute rib fracture if the positive correlation was close to 1 and the negative correlation was close to -1. A larger absolute value of the correlation coefficient indicates that the variable is more important.

As shown in Figure 9, a Spearman correlation analysis was performed to evaluate the association between the radiographic findings and the model-predicted probability of acute rib fracture. Several radiographic components were positively correlated with the model output, suggesting that these features may contribute to increased predicted fracture risk. Among them, the nasogastric tube (r = 0.323), endotracheal tube (r = 0.305), cardiomegaly (r = 0.302), and costophrenic angle blunting (r = 0.272) had the strongest positive associations. Other positively correlated findings included pleural effusion (r = 0.232), fracture (r = 0.205), inflammatory changes (r = 0.193), atherosclerosis (r = 0.165), and pneumothorax (r = 0.107). Additionally, chronic skeletal or degenerative changes such as osteophyte formation (r = 0.092), degenerative joint disease (r = 0.071), and osteoporosis (r = 0.070) demonstrated mild positive correlations, implying that structural bone alterations might be recognized by the model as contextual indicators of rib fragility. Conversely, radiographic features unrelated to acute trauma, including widening of the mediastinum (r = 0.130), vertebroplasty (r = 0.050), and tracheostomy (r = 0.097), showed weak or negligible correlations with the model output.

## Discussion

In this multi-site retrospective study, we developed and validated a deep learning model that predicts CT-confirmed acute rib fractures from chest radiographs and identifies significant associations with the risk of early thoracic complications, including pneumothorax and pulmonary contusion. The model achieved high discriminative performance for acute rib fracture detection, with excellent negative predictive value, and maintained acceptable performance across most clinically relevant subgroups. These findings suggest that CT-anchored AI models applied to routine chest radiographs can reliably exclude clinically significant rib fractures and support nonradiologists in acute care settings.

Several groups have previously applied AI to rib fracture detection on chest radiographs. Specifically, our model's discriminative performance (AUROC 0.874) is quantitatively comparable to prior benchmarks, such as the YOLOv3-based model by Wu *et al*. had the result of AUROC of 0.92^15^ and Huang *et al*. trained deep convolutional neural networks on a large dataset and achieved an AUROC of 0.884 for rib fracture recognition^16^. More recently, Lee *et al*. proposed a Faster R-CNN-based model (Detectron2 framework) that classified both radiographs and localized individual rib fractures; they reported an AUROC of 0.89 and a JAFROC figure-of-merit of 0.76, with a diagnostic performance similar to that of radiologists^11^. A key distinction, however, is that our model was trained using CT-confirmed acute rib fractures as the reference standard rather than radiologists' interpretations of CXRs, which likely reduced label noise and aligned model outputs more closely with the true diagnostic gold standard.

Our findings also complement prior work on CT-based AI for rib fractures. Yao *et al*. proposed a three-step deep learning pipeline for rib fracture detection on chest CT and reported a lesion-level F1-score of 0.89, with radiologist-AI collaboration further improving the sensitivity and reducing the reading time^17^. Tan *et al*. evaluated a commercial deep learning-based computer-aided diagnosis (DL-CAD) system for acute rib fractures on CT and demonstrated substantial gains in sensitivity (from 68.8% to 91.8% for interns and from 86.5% to 94.6% for attendings), along with shorter reading times and improved diagnostic confidence^18^. A recent systematic review and pooled analysis by Hengst *et al*. revealed that CT-based deep learning models for rib fracture detection achieved higher sensitivity than clinicians did overall, particularly for displaced fractures^19^. Taken together with our CXR-based approach, these data suggest that integrating AI across imaging modalities in thoracic trauma workflows has the potential to enhance diagnostic consistency while alleviating radiologist workload.

In contrast to the robust performance observed for pneumothorax and pulmonary contusion, the model's ability to predict pneumonia was suboptimal. This discrepancy is likely driven at least in part by limitations in outcome ascertainment rather than by an absence of signal in the images. Multiple systematic reviews have highlighted that administrative database algorithms based on ICD codes for pneumonia and lower respiratory tract infection are heterogeneous, often poorly validated, and prone to misclassification bias when used for case identification in epidemiological and outcome research^20^, ^21^. Sensitivity estimates are frequently below 80%, and performance varies substantially across coding strategies and clinical settings^20^, ^21^. In our health care system, pneumonia codes are also commonly assigned as provisional admission diagnoses and may subsequently be revised, further weakening the link between rib fractures and pneumonia when ICD codes are used as the sole reference standard. Future work that uses prospectively adjudicated pneumonia endpoints or labels derived from detailed chart reviews and radiological reports will be important to more accurately assess the model's ability to capture this complication.

Our subgroup analyses revealed that the model maintained a high negative predictive value across departments and most demographic subgroups. To further address the potential for shortcut learning driven by clinical severity markers, we performed an ablation analysis (Figure 3). The results demonstrated that the model maintained stable and robust performance even after excluding radiographs with life-support tubes (e.g. endotracheal or nasogastric tubes) and in the outpatient (OPD) cohort where such devices are rare.

Nevertheless, despite the model's technical resilience against these clinical artifacts, its discrimination declined in older adults and in patients with comorbidities, such as diabetes, chronic kidney disease, heart failure, and coronary artery disease. This pattern likely reflects the combined effects of age-related skeletal changes, overlapping cardiopulmonary abnormalities, and imaging artifacts that can obscure subtle fracture lines and early parenchymal changes. Clinically, these findings suggest that a negative model output may be most useful for safely reducing CT utilization in younger, otherwise healthy trauma patients with a low-to-intermediate pretest probability of rib fracture, whereas in frail or multimorbid older patients, CXR-based AI should be considered an adjunct rather than a replacement for CT when clinical suspicion remains high.

It is important to clarify that our model performs rib fracture prediction and complication association rather than direct complication detection. By predicting the probability of acute fractures, the model serves as a robust proxy for stratifying the risk of associated thoracic complications. The strong correlation observed between high predicted fracture risk and the actual incidence of pneumothorax and pulmonary contusion (Figures 7 and 8) validates this descriptive association approach as a clinically useful triage tool.

Methodologically, the use of CT-confirmed labels for both the presence and timing of rib fractures represents a major strength of this study. Prior CXR-based AI models have relied mainly on radiologists' interpretations or manual annotations of the radiographs themselves as ground truth 15, 16 which inevitably incorporate intra- and interreader variability and may be influenced by fatigue and case mixing. By anchoring our labels to CT, we reduce such variability and better approximate the clinical decision standard in trauma imaging. Moreover, we evaluated the model across multiple care settings and clinically meaningful subgroups, providing an initial view of its generalizability within a large, unselected hospital population.

Several limitations should be acknowledged. First, although the model development and tuning were rooted in a tertiary medical center, this was a multi-site study that utilized an independent validation cohort from an affiliated regional branch hospital. However, we acknowledge that both institutions belong to the same healthcare system and may share standardized imaging protocols, equipment, and clinical workflows. Consequently, this evaluation does not constitute a fully independent external validation across different geographic regions or distinct healthcare infrastructures. Nevertheless, we maintain that this validation remains clinically meaningful as it tests the model across different healthcare tiers—specifically, a primary trauma referral site versus a community-oriented regional hospital. These two settings naturally involve distinct patient demographics and trauma severities, providing an initial assessment of the model's generalizability across various levels of clinical practice. Second, CT was not performed for all patients who underwent CXR, so spectrum bias is possible, particularly if clinicians preferentially ordered CT for more severely injured or clinically suspicious cases. Such enrichment of the cohort with higher-severity cases likely inflates diagnostic performance estimates, such as AUC and sensitivity, compared to a broader population with a lower clinical probability of injury. However, we contend that this focus aligns with the model's intended clinical role as a triage safety net in high-pressure environments, where the primary objective is to minimize the risk of missed occult fractures that could lead to significant morbidity. Even if a lower disease prevalence in a general population might further reduce the positive predictive value, the consistently high negative predictive value observed across all subgroups reinforces the model's reliability as a tool for confidently ruling out acute rib fractures. Nevertheless, this study design was a deliberate methodological choice to prioritize the integrity and accuracy of our ground-truth labels. Third, resizing radiographs to 256 × 256 pixels is a prerequisite for the ViT-B/32 architecture in our foundation model framework, which may lead to the loss of subtle fracture details. Unlike traditional CNNs that can process high-resolution patches, this Transformer-based approach prioritizes global semantic representations learned at standardized resolutions. We acknowledge that this constraint may limit the detection of non-displaced or hair-line fractures, highlighting the need for future research incorporating high-resolution Transformer architectures.

Fourth, the reliance on ICD codes for pneumonia outcomes, with its inherent misclassification and timing issues discussed earlier, remains a limitation. Furthermore, in our healthcare system, pneumonia codes are frequently assigned as provisional admission diagnoses and may be revised upon discharge, further weakening the correlation between initial fracture risk and final coding outcomes. While manual chart review or prospective adjudication would offer higher precision, the large scale of our cohort (14,758 patients) rendered such processes impractical for this retrospective investigation. Fifth, we acknowledge that this pilot validation did not include formal multi-rater adjudication, which remains a limitation. Sixth, the number of deaths and several rare complications was too small to support robust modeling of mortality or less frequent adverse events. Finally, we did not assess how clinicians interact with the model in real-world practice or its impact on workflow, diagnostic time, downstream imaging, or patient-centered outcomes; these questions are crucial for implementation and should be addressed in prospective trials.

Future research should therefore focus on multicenter, prospective studies that integrate the model into thoracic trauma care pathways, quantify its effect on CT utilization and time to diagnosis, and evaluate patient safety and clinical outcomes. Combining CXR-based AI with readily available clinical variables—such as the mechanism of injury, pain scores, oxygenation, and comorbidity profiles—may further enhance risk stratification for both fractures and downstream complications. In summary, our deep learning model demonstrates that CT-anchored AI applied to routine chest radiographs can accurately exclude acute rib fractures and identify patients at increased risk of early thoracic complications. If validated prospectively, such tools could help standardize rib fracture assessment, support nonradiology clinicians in high-pressure care environments, and ultimately improve safety and resource allocation in thoracic trauma management.

## Supplementary Material

Supplementary table.

## Funding

This study was supported by funding from the National Science and Technology Council, Taiwan (NSTC 112-2222-E-016-001-MY2 to D.J. Tsai and NSTC 114-2321-B-016-005, NSTC 115-2321-B-016-003 to S.H. Lin), Tri-Service General Hospital, Taiwan (TSGH-B-115025 to T.W. Chen), Cheng Hsin General Hospital, Taiwan (CHNDMU-115-03 to D.J. Tsai).

## Figures and Tables

**Table 1:** Characteristics of the patients in the training, tuning, and validation sets.

Variable	Training	Tuning	Validation	p value
Position				< 0.001
ER	4413 (52.7%)	1798 (53.5%)	1183 (39.2%)	
IPD	1962 (23.4%)	741 (22.0%)	509 (16.9%)	
OPD	1657 (19.8%)	694 (20.6%)	1181 (39.1%)	
PEC	272 (3.2%)	107 (3.2%)	126 (4.2%)	
Unknown	71 (0.8%)	23 (0.7%)	21 (0.7%)	
Age	64.2±18.6	64.5±18.6	59.9±18.5	< 0.001
Gender				0.279
Female	3486 (41.6%)	1433 (42.6%)	1304 (43.2%)	
Male	4889 (58.4%)	1930 (57.4%)	1716 (56.8%)	
DM				< 0.001
	1863 (22.2%)	760 (22.6%)	453 (15.0%)	
HTN				< 0.001
	644 (7.7%)	274 (8.1%)	123 (4.1%)	
CKD				< 0.001
	1997 (23.8%)	779 (23.2%)	323 (10.7%)	
HLP				< 0.001
	2302 (27.5%)	943 (28.0%)	651 (21.6%)	
HF				< 0.001
	923 (11.0%)	418 (12.4%)	172 (5.7%)	
CAD				< 0.001
	1785 (21.3%)	751 (22.3%)	456 (15.1%)	
COPD				0.054
	1489 (17.8%)	638 (19.0%)	503 (16.7%)	
Group of CXR predict acute rib fracture risk				< 0.001
Low risk	7107 (84.9%)	2889 (85.9%)	2729 (90.4%)	
Median risk	919 (11.0%)	343 (10.2%)	213 (7.1%)	
High risk	349 (4.2%)	131 (3.9%)	78 (2.6%)	
Acute rib fracture				< 0.001
No	7,992 (95.4%)	3,213 (95.5%)	2,934 (97.2%)	
Yes	383 (4.6%)	150 (4.5%)	86 (2.8%)	

ER, emergency room; IPD, inpatient department; OPD, outpatient department; PEC, preexamination center; DM, diabetes mellitus; HTN, hypertension; CKD, chronic kidney disease; HLP, hyperlipidemia; HF, heart failure; CAD, coronary artery disease; COPD, chronic obstructive pulmonary disease.
