# Supplementary material for: Computed Tomography-Verified Deep Learning Model for Rib Fracture Prediction and Complication Association from Chest Radiographs
Source: Int J Med Sci. 2026 Jul 22;23(9):2796–809. doi: 10.7150/ijms.131105 (PMC13539916; doi:10.7150/ijms.131105)
Supplement: Supplementary file 1 [file ijmsv23p2796s1.pdf]

Clinical Condition	ICD-9 Codes	ICD-10 Codes
Traumatic Conditions		
Pneumothorax	512.x, 860.x	J93.x, S27.x
Rib fracture	807, 819, 828	S22.x
Pneumonia	480.x–486.x	J18.x, Z87.01
Lung contusion	861.x, 922.1	S27.32x
Comorbidities		
Diabetes mellitus (DM)	250.x	E11.x
Hypertension	401.x–404.x	I10.x–I16.x
Dyslipidemia	272.x	E78.x
Chronic kidney disease (CKD)	585.x	N18.x
Heart failure (HF)	428.x, 398.91, 402.x1	I50.x
Coronary artery disease (CAD)	410.x–414.x, 429.2	I20.x–I25.x
COPD	490.x–496.x	J44.9

Table S1. ICD-9 and ICD-10 codes used for clinical conditions and complications.
